# Adjusting for Resident Rater Leniency or Severity Improves the Reliability of Routine Resident Evaluations of Faculty Anesthesiologists

**DOI:** 10.7759/cureus.86366

**Published:** 2025-06-19

**Authors:** Franklin Dexter, Terrie Vasilopoulos, Brenda G Fahy

**Affiliations:** 1 Anesthesia, University of Iowa, Iowa City, USA; 2 Anesthesiology/Orthopedics and Rehabilitation, University of Florida College of Medicine, Gainesville, USA; 3 Anesthesiology, University of Florida, Gainesville, USA

**Keywords:** clinical competence, educational measurement, logistic regression model, observer variation, performance evaluation, statistical models

## Abstract

Background

The Accreditation Council for Graduate Medical Education (ACGME) of the United States requires all programs to evaluate faculty performance annually. Multiple universities require all faculty to be reviewed annually. These high-stakes evaluations should be reliable. When one anesthesiologist is said to perform better than another, there should be neither frequent Type I errors (i.e., an anesthesiologist is determined to perform better or worse than average when their performance is average) nor Type II errors (i.e., failure to detect above or below average performance)*. *We investigated the generalizability of the finding that if adjustment is not made for rater leniency/severity, results will be statistically unreliable.

Methods

University of Florida 11-item evaluations were sent on Mondays, over the 2018-19 academic year. 108 ratees (anesthesiologists) had 3302 evaluations by 85 raters (resident physicians). The replicability of the results was assessed by making a comparison with previously published findings from the University of Toronto and the University of Iowa.

Results

As observed at the University of Toronto, there was greater heterogeneity of scores among raters than among ratees (raters’ eta-squared 0.40; ratees’ 0.22). As observed at the University of Iowa,the Florida rater leniency/severity of scores could not validly be modeled based on a normal distribution, because the distribution of each rater’s mean among raters was not normally distributed (Shapiro-Wilk W = 0.90 (P = 0.00002) among the 75 raters with ≤9 evaluations). Likewise, matching Iowa,Florida’s distribution of each ratee’s mean among ratees was not normally distributed (W = 0.91 (P = 0.00001) among the 94 ratees with ≤9 evaluations). In contrast, treat evaluations with all items scored the maximum as having a value of 1, otherwise 0. As for Iowa,Florida’s corresponding probability distributions of logits were normally distributed (W = 0.99 (P = 0.90) among raters and W = 0.98 (P = 0.09) among ratees, respectively). Rater leniency/severity remained large in the logit scale, with an intraclass correlation coefficient of 0.55. In the original scale, 0/108 ratees had performance that differed significantly from the grand mean of 4.63, using a P < 0.01 criterion. The alternative analysis approach adjusted for the raters’ leniency/severity. Seven ratees were significantly below average (P ≤ 0.0048) and 17 above average (P ≤ 0.0086). Because statistical assumptions were satisfied, analysis in the original scale had a 22% (24/108) false negative rate, like the 21% observed previously at the University of Iowa.

Conclusions

Routine evaluations of faculty anesthesiologist ratees by anesthesiology resident raters give statistically unreliable results, falsely categorizing performance, unless analyses are adjusted for the covariates of raters. The need for adjustment found with the University of Florida data matches the need for this type of adjustment found at the University of Iowa and the University of Toronto*.* Thus, this adjustment for raters’ leniency/severity appears to be a general finding for rater/ratee routine evaluations.

## Introduction

The Accreditation Council for Graduate Medical Education (ACGME) of the United States requires that all programs evaluate each faculty member’s performance “as it relates to the educational program at least annually” [1]. At the University of Iowa and at the University of Florida, all faculty must be reviewed annually [2,3]*.* For these high-stakes decisions, evaluations should be both valid and reliable [4-7]. In this context, valid means that the evaluation is measuring the intended content. When anesthesiologists supervise anesthesiology residents or nurse anesthetists [4-7], multiple studies from several hospitals and nationwide have shown the validity of the de Oliveira Filho supervision instrument (see Discussion [5,8-16]). The quality of clinical supervision can be evaluated using the supervision instrument repeatedly for many years [13,14,17]. The focus of the current study, however, is different: reliability.

When one anesthesiologist is said to perform better than another using the chosen instrument, there should neither be frequent Type I errors (i.e., anesthesiologist determined to perform better or worse than average when performance is average) nor Type II errors (i.e., failure to detect above or below average performance) [6,7]. A reliable instrument differentiates appropriately when differences exist, but does not do so falsely. In multiple earlier studies from the University of Iowa, anesthesiologists’ quality of clinical supervision and nurse anesthetists’ work habits were evaluated reliably [6,7,13-15,18,19]. However, such evaluation of reliability included control for the leniency/severity of raters [6,7,20].

Leniency versus severity is a continuum, with lenient raters assigning higher scores and strict raters assigning lower scores [6,7,15-23]. At the University of Iowa, the individual raters were included in the statistical models for evaluating quality of supervision or work habits because otherwise their implicit or explicit biases caused unreliable assessment of the ratees [6,7,15,24]. Specifically, using 2015 data, failure to adjust for rater leniency when using the supervision instrument [4] would have reduced the odds of detecting outlier performance 6.67‑fold, P = 0.0008 [6]. Using 2018 data, failure to adjust would have resulted in a 21% misclassification rate of anesthesiologists as being below average, above average, or having average performance (15/73) [7].

Unlike assessments of validity [4,5,8-16], considered in the Discussion, the findings for reliability [5-7,19,21] were from one organization, the University of Iowa. Generalizability of the findings for reliability [5-7,19,21] was expected because the American Board of Anesthesiology makes such adjustments for oral examination scoring [25]. However, such generalizability of results for reliability had been untested for routine (e.g., daily or weekly) evaluations. Then, in 2023, the University of Toronto reported similar findings for faculty evaluation of residents using the Anesthesia Clinical Encounter Assessment [26]. When faculty anesthesiologists evaluated resident physicians as “entrustable” or not, the estimated variance among raters (2.63 logit units) was approximately five-fold greater than the variance among the ratees (0.52 logit units) [26]. Therefore, we took advantage of a natural experiment for the current study of reliability. The University of Florida previously analyzed its faculty anesthesiologists using a single 11‑item instrument. We used these data to test the generalizability of the finding from the University of Iowa that, when routinely evaluating faculty anesthesiologists, if adjustment is not made for rater leniency/severity, results will be statistically unreliable, causing inaccurate categorization of anesthesiologists’ performance as below or above average [6,7,15].

## Materials and methods

The University of Florida Human Research Protection Program determined that this project qualifies for exemption under the United States regulations that govern human subjects research, including no requirement for consent (protocol # ET00041582, approval date: 10 June 2024).

We refer to evaluations using two different instruments, the de Oliveira Filho supervision instrument used in earlier studies (Table 1) [5,8-16] and the University of Florida's instrument used in the current article (Table 2). The evaluations studied from the University of Florida Gainesville campus were made of anesthesiologists providing care in operating rooms (72%), acute pain medicine (14%), chronic pain (6%), critical care medicine (5%), and obstetrics (4%). All University of Florida evaluations were sent on Mondays, over one academic year, July 2018 through June 2019. The date range was chosen as the most recent year for which the anesthesiology residents doing the ratings had completed the program. Just as for the University of Iowa’s evaluation of supervision quality (Table 1) and work habits [6,13,14,21], ratees received summary measures using one year of data, such that there was no possibility that a ratee could validly associate their performance evaluation with how they may have evaluated a rater. Furthermore, earlier studies showed no relationship (i.e., no retaliation) between anesthesiology residents' and faculty anesthesiologists' evaluations of each other [27].

**Table 1:** Earlier findings of the validity of evaluating anesthesiologists’ quality of clinical supervision using the de Oliveira Filho et al. instrument The methods used in the current study rely on the earlier findings of the validity of the de Oliveira Filho et al. supervision instrument [4,13,14]. The first word in the second column is bolded to explain the sequencing of the eight rows. The populations labeled "USA" were from a national survey of anesthesiology residents performed by de Oliveira et al. [8].

Row	Findings of validity	Population	Year	Reference
1	Inverse association between scores and anesthesia residents’ reports of: a) “mistakes that [had] negative consequences for the patient,” b) “medication errors in the last year,” and c) “errors with potential negative consequences to patients”	USA	2015	[8]
2	Inverse association between scores and operating room times (i.e., higher quality of supervision associated with briefer procedure-adjusted operating room times)	Iowa	2025	[13]
3	Inverse association between scores and nurse anesthetists’ reports of hardly ever seeing the anesthesiologist during the day	Iowa	2015	[10]
4	Positive associations between scores and evaluations using assessments of teaching quality	Iowa	2013, 2020	[9,15]
5	Positive association between scores and anesthesia residents’ reports of the “overall perceptions of patient safety,” “nonpunitive response to errors,” “communication openness,” “feedback and communication about error,” and suitability to care for family	Iowa, USA	2013, 2015	[8,9]
6	Positive association between scores and comments made about the anesthesiologists treating others disrespectfully	Iowa	2016	[11]
7	Absence of association of scores with raters’ age, gender, estimated hours worked per week, year of training, weeks in rotation, size of residency class, or hours worked with the anesthesiologist that day	Iowa, USA	2013, 2015, 2024	[8,12,16]
8	Absence of association of scores with cases’ time of day, physiological (anesthetic) complexity, or surgical specialty, patients’ ages, or anesthesiologists’ other rooms that day	Iowa	2024	[16]

**Table 2:** The 11 items in the University of Florida Gainesville instrument that were originally analyzed by using their mean The 11 items analyzed with each evaluation at the University of Florida are listed. Each item had five radio buttons without numbering. The department interpreted five as “outstanding,” four as “above average,” three as “average,” two as “below average,” and one as “unacceptable.” This work, created by the University of Florida, was licensed under the Creative Commons Attribution 4.0 International License, without revisions.

Item #	Text	Mean (SD)	% (fraction) equal to 5
1	Clear/organized	4.62 (0.60)	67% (2224/3301)
2	Enthusiastic/stimulating	4.61 (0.63)	69% (2271/3298)
3	Supportive of me/had rapport with me	4.63 (0.61)	70% (2316/3295)
4	Clinically competent/knowledgeable	4.69 (0.54)	73% (2410/3282)
5	Provided timely, constructive feedback	4.62 (0.62)	69% (2247/3276)
6	Explained basis for decisions/actions	4.63 (0.60)	70% (2286/3276)
7	Responded to student-initiated learning topics	4.63 (0.60)	69% (2203/3183)
8	Emphasized comprehension of concepts rather than merely factual recall	4.64 (0.59)	70% (2249/3223)
9	Overall teaching effectiveness	4.61 (0.62)	68% (2238/3272)
10	Degree to which this faculty member is a role model for professionalism, including compassion for patient and demonstrating respect for the operating room team.	4.63 (0.61)	69% (2267/3247)
11	I would actively seek this faculty member to care for me or a family member.	4.64 (0.61)	70% (2308/3281)
Mean	(Mean of the 11 items)	4.63 (0.55)	58% (1914/3302)

Our research plan was to reproduce the calculations of reliability performed using the de Oliveira Filho supervision instrument at the University of Iowa (Table 1) [5-7,19,21] using the University of Florida evaluations (Table 2). We used Stata v18.5 (StataCorp, College Station, USA). By doing so, generalizability would be assessed both in terms of institution and instrument. The Stata output for all analyses is available at https://doi.org/10.25820/code.007640 

Evaluations using the University of Florida instrument (Table 2) were distributed via New Innovations software, with a link provided to the evaluation (Uniontown, USA). Requests were emailed every three days once delinquent, for a total of five times. Evaluations had 11 specific items plus an overall score requesting comments.

We analyzed the 11 items of the University of Florida instrument (Table 2). Each item was scored from 1 to 5 (Table 2). The 95% confidence interval for Cronbach's alpha was calculated using the method described by Feldt et al., based on the smallest of the observed sample sizes of evaluations among items (Table 2) [28,29]. Using the smallest observed sample size of N = 3183, the confidence interval was deliberately conservative (i.e., wide). P < 0.05 was treated as statistically significant.

Initial analyses using the original scale used by raters for their scores

To compare results with those from the University of Toronto using the Anesthesia Clinical Encounter Assessment [26], eta-squared and their 97.5% simultaneous confidence intervals were calculated using fixed effects two-way analysis of variance for rater and ratee categorical effects, with dependent variable of the mean of the one to five scores among items for each evaluation (Table 2). Eta-squared is a unitless measure of effect size for analysis of variance. Because eta-squared is a biased estimator, we also include omega-squared, but the latter without a confidence interval.

Shapiro-Wilk tests were used to compare distributions of the means of scores among raters and among ratees to normal distributions. The reason for comparing each rater’s mean among raters, and each ratee’s mean among ratees, was that the model for means to follow normal distributions matches the random effect formulation used in mixed effects models. If they do not follow normal distributions, as assumed, then posterior means of the random effects will be biased, causing Type I or II errors in the classification of the ratees. Shapiro-Wilk tests were performed among raters and ratees, each with at least nine evaluations, so the means were relatively precise. Analyses used the category of raters as the sole covariate because dozens of other potential covariates were nonsignificant (e.g., raters’ age, gender, and rotation) [8,16]. Performance changes over time [6,16,20,30], but that was not applicable as our analyses were limited to one such period (Table 2).

Analyses after transformation of total scores to the logit scale

Treat each evaluation binary: one if all the evaluation’s items are scored the maximum, and zero otherwise. The logit for a rater, or ratee, equals the natural logarithm of a ratio, with the numerator being the proportion of evaluations with all items equal to the maximum, and the denominator equaling one minus that proportion. If the internal consistency were large, as hypothesized (above), we expect from earlier results with the nine-item de Oliveira Filho et al. supervision scale that approximately 80% of evaluations would have all items equal to the maximum [20]. A high proportion (≈0.80) was expected because these were routine evaluations of ratees who were licensed professionals while they cared for anaesthetized patients at a tertiary hospital, with trainees (Table 1) [12]. We have shown previously that the same applies to the nurse anesthesia work habits scale [18,24] and evaluations of Uber riders [31,32].

The magnitude of rater heterogeneity in the logit scale was quantified using the intraclass correlation coefficient, estimated using an intercept-only mixed effects logistic regression model. The area under the receiver operating characteristic curve was also calculated, using the corresponding fixed-effect logistic regression model, again with raters only.

A suitable sample size, to reliably know that the means or the logits followed a normal distribution, was to have at least 40 raters and ratees [3], a condition satisfied for the current study. The criterion of 40 raters or ratees results in 80% statistical power for the Shapiro-Wilk test to detect deviation from a normal distribution [33]. In addition to P > 0.05 showing adequate fit to normal distributions, the W statistic being close to its asymptotic maximum of 1 shows adequacy of fit.

To test the probability distribution of the logits, some raters with at least nine evaluations had all evaluations equal to the binary of 1 (i.e., all items scored the maximum). Each was given a logit score equal to 1 minus 0.5 divided by their number of evaluations plus 1 (i.e., continuity correction). Similarly, those raters with all evaluations equal to the binary of 0 (i.e., no evaluations with all items scored the maximum) were given a logit score equal to 0.5 divided by their number of evaluations plus 1. For the subsequent mixed effects logistic regression, these raters were perfectly predictive, causing complete separation and were thus dropped by the regression [7,15,24,31]. (Note that this behavior is not a limitation, but desired, integral to logistic regression, and used at the University of Iowa to provide feedback to raters on the quality of their evaluations [7,31].)

Comparisons of ratees in original and logit scales

Statistical significance of each rater’s evaluations in the original scale versus the grand mean was evaluated using one-group Student t-tests (i.e., using each rater’s mean and standard deviation). P < 0.01 was treated as statistically significant to adjust, in part, for the multiple comparisons. Previously, using data from the University of Iowa with the de Oliveira Filho et al. supervision instrument [4], 30/83 ratees had low mean scores [6]. Among those 30, there were 8/30 significantly below average by Student t‑tests [6]. For eight of the eight ratees, analyses in the logit scale detected that below average performance [6]. (These previous results are used in the current study's results, below.) However, analyses in the logit scale also detected 13/30 others with below-average clinical supervision [6]. Specifically, to adjust for raters’ leniency/severity of scores, the raters were entered as fixed effects into the mixed effects logistic regression model [6,7,13,21,24]. The ratees were a random effect [1]. Mixed effects models were used, with ratee as a random effect, because with ratee as a fixed effect, there is a substantial false positive rate of apparent outlier ratees [24,34]. The mechanism is that logistic regression with rater and ratee as fixed effects has small standard errors for multiple fixed effects with small sample sizes in the numerator [24]. The posterior means of the random effects and their standard errors (i.e., empirical Bayes estimates) were simulated using 30 quadrature points [6,13,24]. The estimated odds ratios are the exponential of these means. Odds ratios >1 show evaluation performance better than average, and vice versa. Odds ratios are used because they are calculated with standard errors of the empirical Bayes random effects estimators. The incremental information about the ratees is the random effects. Results are not reported as predicted mean proportions of evaluations for each ratee with all eleven items scored five (Table 2) because there are also different fixed effects (i.e., raters). The Z‑statistic for each ratee was the ratio of the absolute value of the rater’s posterior means to the rater’s corresponding standard error. The inverse of the standard normal distribution was taken for the Z-statistic, and statistical significance was identified for the ratee if P < 0.01 [6,7,13].

## Results

Among the 3306 evaluations for the academic year at the University of Florida, Gainesville, using their instrument (Table 2), there were four evaluations with more than three missing scores among the 11 items, leaving 3302 completed evaluations that were analyzed. There were 108 ratees. There were 85 raters, from postgraduate year one (19), two (23), three (24), and four (19). The 11 evaluation items were internally consistent, with a Cronbach's alpha 0.980 (95% confidence interval 0.979 to 0.981). There were 58% of evaluations (1914/3302) with the maximum score of 5 for all items scored. The second most common response pattern was all items being 4, and then three, two, or one item(s) being 4 (i.e., as seen at the University of Iowa for both the supervision and work habits instruments, there was not a monotonic frequency distribution; see Supplemental at https://doi.org/10.25820/code.007640 for all percentages) [6,13,15,24]. Among the 9180 possible pairings of ratee and rater, where 9180 = 108 × 85, there were 2556 observed, among which 60% were one evaluation (1989/2560).

Initial analyses using the original scale used by raters for their scores

As observed at the University of Toronto when they used the Anesthesia Clinical Encounter Assessment [26], the University of Florida’s instrument (Table 2) resulted in significantly greater heterogeneity of scores among raters than among ratees (raters’ eta-squared = 0.40, 97.5% confidence interval 0.36 to 0.41, omega-squared = 0.39; ratees’ eta-squared = 0.22, 0.16 to 0.22, omega-squared = 0.19); N = 3302 evaluations.

**Figure 1:**
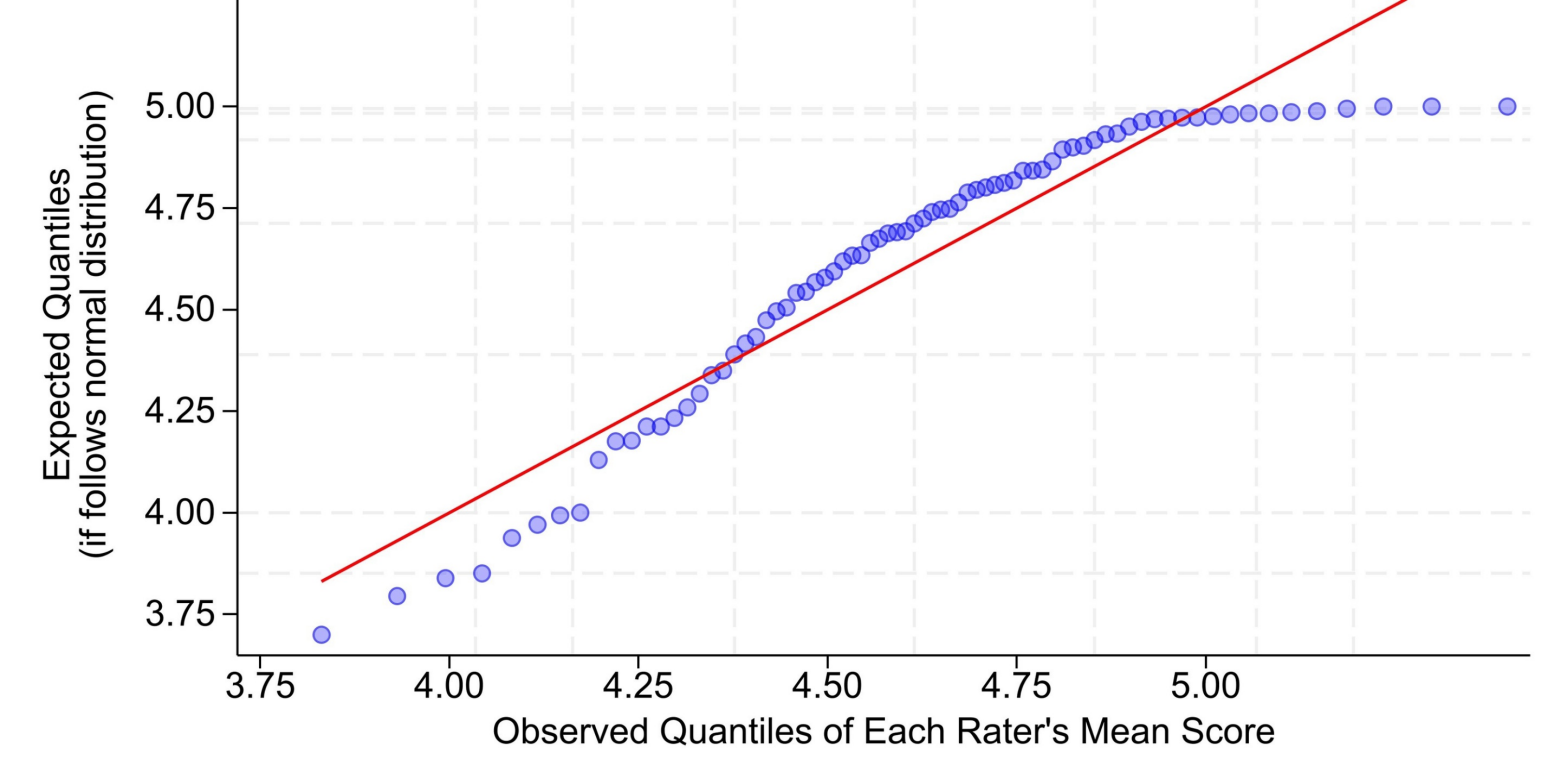
Quantile-quantile (Q-Q) normal distribution plot for the rater’s mean scores Along the horizontal axis are plotted the observed mean evaluation scores for each of the 75 raters with at least nine evaluations. Because the mean among evaluations of each evaluation’s mean score equals the overall mean, the value plotted on the horizontal axis is literally the mean of all the 1s to 5s (Table 2) scored by each rater. Next, calculate the percentile for each rater’s mean score compared to all other raters’ mean scores. The grid lines are 5, 10, 25, 50, 75, 90, and 95 percentiles. The vertical axis shows the expected location on the graph if the percentiles followed a normal distribution. There would be a straight line, as shown by the red line. The graph shows two results. First, the data markedly deviate from a normal distribution, as quantified with the Shapiro-Wilk W = 0.90 and P = 0.00002. Second, the raters deviate markedly in their leniency/severity of scores, based on the range on the horizontal axis. This variability among raters is quantified by the eta-squared, omega-squared, intraclass correlation, and area under the receiver operating characteristic values given in the results.

Satisfying probability distribution statistical assumptions is important because faculty evaluations are high-stakes (e.g., used for promotions) [4,7]. As observed with University of Iowa data (Table 1) [6,20], both the supervision and work habits instruments [24], the University of Florida’s rater leniency/severity of scores with their instrument (Table 2) could not validly be modeled based on a normal distribution, because the distribution of each rater’s mean among raters was not normally distributed (Shapiro-Wilk W = 0.90, P = 0.00002, among the 75 raters each with at least nine evaluations; Figure 1). Likewise, matching the University of Iowa’s findings with both supervision and work habits instruments [6,24], the University of Florida’s distribution of each ratee’s mean among ratees was not normally distributed (W = 0.91, P = 0.00001) among the 94 ratees, each with at least nine evaluations.

Analyses after transformation of total scores to the logit scale

As found for the University of Iowa for supervision and work habits instruments [6,24], the University of Florida’s data with their instrument had corresponding probability distributions of the logits were normally distributed (W = 0.99, P = 0.90, among raters and W = 0.98, P = 0.09, among ratees, respectively). Also, as for the University of Iowa, rater leniency/severity remained large in the logit scale, with an intraclass correlation coefficient of 0.55, and had a 95% confidence interval of 0.45 to 0.64. The area under the receiver operating characteristic curve was 0.82. Probability plots are in the supplemental content.

Comparisons of ratees in original and logit scales

Each of the 108 ratees’ means and standard deviations in the original scale were used for comparison by Student’s one-group t-test with the N = 3302 evaluations’ grand mean score of 4.63. None of the 108 ratees had performance that differed significantly using a P < 0.01 criterion. (The result of zero ratees having below or above average performance would be the same with formal adjustment to control the false discovery rate at P < 0.05.) There was one ratee above average with P = 0.024. For the other ratees, all P ≥ 0.100.

The alternative analysis approach used the 73 raters, both with some evaluations having a mean of the 11 items < 5.00 and some evaluations having all items scored five (see Methods) [7,24,31]. Adjusting for the 73 raters’ leniency/severity and using a P < 0.01 criterion for statistically significant, there were seven ratees significantly below average (all P ≤ 0.0048) and 17 ratees significantly above average (all Ps ≤ 0.0086) [35,36]. Because statistical assumptions of the mixed effects regression were satisfied, the analysis in the original scale had a false negative rate of 22% (24/108). (The comparable result from the University of Iowa with the supervision instrument [4] was 21% (15/73) [7]). The seven ratees below average, using the mixed effects model, had pooled means in the original scale of all items ranging from 3.72 to 4.59 (i.e., just slightly less than the grand mean of 4.63) (Table 2). Likewise, the 17 ratees above average had pooled means ranging from 4.70 (i.e., slightly larger than the grand mean) to 4.96. That pattern also matched the University of Iowa results with the supervision instrument [6].

The preceding analyses with the University of Florida’s instrument used unadjusted two-sided P < 0.01 as the criterion for statistical significance for comparison of results to those from the University of Iowa with supervision and work habits instruments [6,7,13-15,24]. When repeated using Benjamini, Krieger, and Yekutieli’s step-up adjustment method to maintain the false discovery rate < 0.05, even more (38) ratees were significant outliers [35,36], showing the reliability of the finding that ratees differed significantly. A smile plot is shown in the supplemental content.

## Discussion

The current study's results match those from the University of Iowa when anesthesiologists’ quality of clinical supervision and nurse anesthetists’ work habits were evaluated [5-7,20,24]. At the University of Florida with their instrument (Table 2 questions), anesthesiologists cannot accurately be differentiated as above/below average performance using analysis in the original scale (Table 2 second column, means). For all three combinations, plus the University of Toronto applying the Anesthesia Clinical Encounter Assessment, rater leniency/severity has a larger magnitude than the variation among ratees [26]. Summary measures do not follow normal distributions. Evaluation performance behaves in practice as binary, all items equaling the maximum or not. Controlling for rater leniency/severity, ratees can be significantly differentiated. Statistical assumptions are satisfied, as appropriate for these high-stakes assessments, and used to show that the unadjusted analyses’ results were Type II errors.

Before the current study, we knew, from the University of Toronto, that variability among faculty raters’ scores for entrustable professional activities exceeded variability in performance among the resident ratees [26]. Likewise, we knew, from the University of Iowa (Table 1), that unless the leniency/severity of the individual raters (e.g., anesthesiology residents) was controlled statistically, implicit or explicit bias caused imprecise and unreliable assessment of the ratees (e.g., anesthesiologists) [6,7,15,24]. The current study adds to both studies by using data from the University of Florida with a fourth instrument (Table 2) and finding generalizability. However, the consequences differed, providing context for readers. At the University of Iowa, raters (e.g., residents) and ratees (e.g., anesthesiologists) pairwise daily assignments (i.e., to individual cases) significantly differed from random (all studies’ P < 0.00001) [5,19,21]. The consequence was that imprecision and lack of reliability resulted not just in finding ratees as being average when their performance differed from average (Type II errors), but also false classification of ratees as outliers (Type I errors) [6,7]. That was not so at the University of Florida, with how they used their instrument, because ratee-rater pairings were seemingly random. In their data, 78% of the rater and ratee evaluations were for only one occasion (i.e., week) working together the entire year.

Another hospital implementing our results would not need to repeat our work. Rather, rely on our results by interpreting their daily or weekly evaluations using mixed effects logistic regression with empirical Bayes estimates for the ratees' (e.g., anesthesiologists’) effects [6,24]. We have provided the relevant two lines of Stata computer code in the supplemental labeled “mixed effect model,” along with supportive code depending on desired reports (e.g., confidence limits). There is also an online lecture showing examples [37]. The same approach can be used for evaluating pain medicine physicians [15], overall anesthesia department quality of clinical care [20], and nurse anesthetists’ work habits [7,13,14,16-18,21,24,31]. Importantly, for departments that rely principally or solely on qualitative comments, doing so does not mitigate rater bias; rather, it obfuscates the implicit or explicit bias that inevitably is present [31].

Previously, simulations were performed to compare hospital performance, showing that mixed effects logistic regression is best used [34]. The dependent variable was binary, like in the current study [34]. The ratees, hospitals, or anesthesiologists, were the random effect, like in the current study [34]. The conclusion that adjustment should be made for raters’ leniency/severity is comparable to finding that the patients cannot be treated as interchangeable and equivalent among hospitals. To the extent that seems obvious about patients and hospitals, the same is shown here for rating anesthesiology residents. Residents are not interchangeable (Figure 1).

Conceptually, the problem of raters’ leniency/severity could be avoided by evaluating faculty anesthesiologists quantitatively, but not based on residents’ surveys. There are two limitations to this approach. First, trainee evaluations of faculty are mandatory for national (US) residency accreditation [1]. Second, these evaluations can be performed using the de Oliveira Filho et al. supervision instrument, which has construct, content, convergent, discriminant, and predictive validity (Table 1) [5,8-13,16]. In contrast, comparing among anesthesiologists based on undesirable clinical and management outcomes has so far been found to be invalid or unreliable, those endpoints including postoperative patient satisfaction [38-40], post-anesthesia care unit pain scores [41], prolonged times to tracheal extubation [42], hypotension during induction of anesthesia [43], intensive care unit admission after ambulatory surgery [44], postoperative length of stay [14], patient mortality or major postoperative complications [45], and practitioner’s unscheduled absences from work [46].

Limitations

The current study's results used the University of Florida's instrument (Table 2), which was neither the de Oliveira Filho et al. unidimensional supervision instrument with multiple validation studies (Table 1) [5,8-13,16] nor the System for Evaluating Teaching Qualities multidimensional questionnaires [47,48]. We recommend that readers use instruments with known validity (Table 1) [5,8-13,16,18,26,47,48]. Nevertheless, Table 2 was used for years. Raters completed the evaluations. Ratees received summary reports. Yet, just like for valid instruments used for regular anesthesia evaluation [13,18,26], our results show that rater leniency/severity was large (Figure 1), as quantified by eta-squared and omega-squared (i.e., relative to variability among ratees), intraclass correlation coefficient, and area under the receiver operating characteristic curve.

Another feature of the current study was that there is no information on response rates, or characteristics of the raters (other than their clinical year of training) or ratees (other than being their faculty), unlike earlier studies of the University of Iowa, Mayo Clinic, and nationally [5,6,8,9,11-13,16]. That was necessary for the availability of the data. Therefore, while the current study was performed to examine the generalizability of studies from the University of Iowa [6,7,15,24] and the University of Toronto [26], and did so successfully, we recognize that future investigators will be limited in considering how our studied population could have affected results.

Although we used resident evaluations of faculty in the manuscript (Table 2), we are not inherently studying the faculty evaluation by residents. For example, using mixed effects logistic regression to adjust routine evaluations for rater severity/leniency was originally developed and tested for nurse anesthetist ratees and anesthesiologist raters using the work habits scale [24]. Rather, we used the dataset from the University of Florida with a fourth instrument to demonstrate further the general need to correct raters for leniency/severity. We were able to replicate the need to correct for rater leniency despite using a novel and unvalidated 11-item evaluation form (Table 2). We now have three examples using different instruments, different rates/ratees, and different institutions (the University of Iowa, the University of Toronto, and the University of Florida) that show the same need for this correction. All three sites also showed the mathematical problem of separation, which refers to raters who give every ratee the maximum score on all items, or vice versa [6,24]. Subsequent development using the work habits instrument revealed that the information content of raters’ evaluations can be quantified using binomial entropy [17,31]. Those results are used for regular email feedback to resident and anesthesiologist raters at the University of Iowa [31,37].

## Conclusions

Routine evaluations of ratees (e.g., faculty anesthesiologists) by raters (e.g., anesthesiology residents) have given statistically unreliable results at three University medical centers using four instruments, falsely categorizing performance unless analyses are adjusted for the covariates of raters. Adjustments for the raters should be made using multivariable linear mixed effects methods shown to have their assumptions satisfied (e.g., following the Stata code in our supplemental content). Once done, the ratees' clinical performance is differentiated reliably.
